# A randomised controlled trial of a smoking cessation intervention delivered by dental hygienists: a feasibility study

**DOI:** 10.1186/1472-6831-7-5

**Published:** 2007-05-02

**Authors:** Vivian I Binnie, Siobhan McHugh, William Jenkins, William Borland, Lorna M Macpherson

**Affiliations:** 1 Glasgow Dental Hospital and School, 378 Sauchiehall St, Glasgow, UK; 2 Department of Biochemistry, Gartnavel Hospital, Glasgow, UK

## Abstract

**Background:**

Tobacco use continues to be a global public health problem. Helping patients to quit is part of the preventive role of all health professionals. There is now increasing interest in the role that the dental team can play in helping their patients to quit smoking. The aim of this study was to determine the feasibility of undertaking a randomised controlled smoking cessation intervention, utilising dental hygienists to deliver tobacco cessation advice to a cohort of periodontal patients.

**Methods:**

One hundred and eighteen patients who attended consultant clinics in an outpatient dental hospital department (Periodontology) were recruited into a trial. Data were available for 116 participants, 59 intervention and 57 control, and were analysed on an intention-to-treat basis. The intervention group received smoking cessation advice based on the **5A**s (ask, advise, assess, assist, arrange follow-up) and were offered nicotine replacement therapy (NRT), whereas the control group received 'usual care'. Outcome measures included self-reported smoking cessation, verified by salivary cotinine measurement and CO measurements. Self-reported measures in those trial participants who did not quit included number and length of quit attempts and reduction in smoking.

**Results:**

At 3 months, 9/59 (15%) of the intervention group had quit compared to 5/57 (9%) of the controls. At 6 months, 6/59 (10%) of the intervention group quit compared to 3/57 (5%) of the controls. At one year, there were 4/59 (7%) intervention quitters, compared to 2/59 (4%) control quitters. In participants who described themselves as smokers, at 3 and 6 months, a statistically higher percentage of intervention participants reported that they had had a quit attempt of at least one week in the preceding 3 months (37% and 47%, for the intervention group respectively, compared with 18% and 16% for the control group).

**Conclusion:**

This study has shown the potential that trained dental hygienists could have in delivering smoking cessation advice. While success may be modest, public health gain would indicate that the dental team should participate in this activity. However, to add to the knowledge-base, a multi-centred randomised controlled trial, utilising biochemical verification would be required to be undertaken.

## Background

Whilst the general health concerns of tobacco use are well known, the detrimental effect of tobacco use on the mouth is less often acknowledged. Oral problems can vary from aesthetic concerns, such as staining of the teeth and halitosis, through to periodontal disease, and more serious conditions such as potentially malignant lesions and oral cancer. There is a well-demonstrated dose-response relationship between tobacco use and risk of developing oral cancer, with the risk increasing significantly with the number of cigarettes smoked and the duration of smoking [1]. Individuals who smoke and do not drink alcohol have a two to four-fold increased risk of developing oral cancer [2,3]. Globally, oral cancer is the sixth most common malignant tumour for both genders [4].

The strong links between cigarette smoking and periodontal disease are also well established, with smokers being approximately three times more likely than non-smokers to have periodontitis [5]. The relationship is even stronger among those more severely affected by the disease. Consequently, smokers tend to have more bone loss and fewer teeth than non-smokers [6].

While the oral problems caused by smoking legitimise the involvement of dental team members in the provision of tobacco cessation advice, it is also important that dental professionals take a holistic approach to health promotion, and provision of smoking cessation advice is an example of this. There is now an increasing interest in the role that the dental team can play in helping their patients to quit smoking.

The Cochrane Library has a number of tobacco-related reviews addressing the issue of physician and nurse-delivered smoking cessation advice [7,8]. The evidence relating to tobacco cessation and the dental team has recently been published; however, of the six studies included in the review, five are associated only with smokeless tobacco [9]. The review concludes that, currently, insufficient evidence exists to make recommendations about the effectiveness of tobacco cessation interventions in a dental setting for cigarette smokers. Of the very limited number of studies in the UK to date, none have utilised a randomised controlled trial design with biochemical verification of cessation. The aim, therefore, of this trial was to examine the feasibility of undertaking an RCT smoking cessation intervention, delivered by trained dental hygienists, in a cohort of periodontal patients attending an outpatient dental hospital department.

## Methods

Having obtained ethical approval from the local Dental Ethics committee, participants were recruited from a cohort of new patients (smoking >10 cigarettes per day) attending Periodontology consultant clinics at a dental hospital, and referred for treatment to the staff dental hygienists. As part of normal professional practice, all new patients received information on the role that tobacco plays in periodontal disease and 'very brief' advice to quit smoking by the examining consultant. Recruitment was by the consultant, who explained the nature of the trial, and gave the patient an information sheet. Informed consent was obtained from the patients prior to participation in the trial. It was made explicit that patients would not necessarily be allocated to receive further advice and help, in addition to their periodontal treatment. Exclusion criteria included those aged below 18 years, those already in receipt of nicotine replacement therapy or currently undergoing smoking cessation therapy or patients with complex medical histories. This study encompassed only cigarette smokers, as it is this group of tobacco users who constitute the vast majority of patients attending for periodontal treatment in this dental hospital setting.

At baseline, socio-demographic information was collected from the participants as well as information on nicotine dependence, as measured by the Fagerstrom Test for Nicotine Dependence [10]. Motivation to quit was also determined using a questionnaire based on 'Stage of Change' [11]. Information on lifetime exposure, as measured by pack-years, was also collected, as was participants' current exposure to smoke at home and work.

The baseline level of smoking was measured by the use of unstimulated salivary cotinine (COT) and level of carbon monoxide (CO) in an exhaled air sample. Cotinine is widely accepted as the most accurate means of measuring tobacco exposure [12]. Cotinine levels were measured using ELISA immunoassay kits (Cozart UK). The detailed methodology for unstimulated salivary sample collection has been published previously [13]. However, carbon monoxide monitoring is the most widely used method of measuring cigarette consumption, and can be useful in determining the smoking status of a patient who is taking NRT. In addition, CO levels can also be used as a motivational tool. The CO monitors used were picoSmokelysers (Bedfont Scientific, UK).

The randomisation process was set up by the project statistician (SM) and was implemented independently from the recruitment process. After a patient was recruited into the study by a consultant, the patient's name was transcribed into a log book, which contained sequential patient log numbers and against each, the allocated hygienist. Having allocated the patient to a hygienist, the patient was then allocated to either intervention or control group using the minimisation method (with weighted randomisation) [14,15]. The allocation to the group was balanced by sex, age (<34, 35–44, 45–54 >55), level of deprivation (as measured by Carstairs Deprivation Index, low/moderate/high DEPCAT [16]), level of nicotine dependence (<6, ≥6) and modified 'Stage of Change' (not interested, concerned about their smoking and wanting to stop).

Those participants allocated to the intervention group received, in addition to their clinical care, smoking cessation advice delivered by one of three staff dental hygienists. These dental hygienists had undergone training, including smoking cessation sessions (1 day equivalent, which covered epidemiology of tobacco use, nicotine dependence, basic smoking cessation skills, supporting the smoker), and training in NRT (1/2 day, which covered nicotine withdrawal, use of NRT products, relevant clinical guidelines) and trial methodology (1/2 day, which covered use of questionnaires in data collection, **5A**s methodology, salivary and CO sampling).

The model of advice used was the **5A**s [17]. This structured advice is based upon **ask/advise/assess/assist/arrange **follow-up for the patient and has been used in a number of settings, such as general medical practice [18]. A customised protocol with particular emphasis on oral health aspects was developed for use by the dental hygienists [19]. As part of the 'assist' phase, the intervention participants, if they so wished, received free nicotine replacement therapy (NRT) in the form of patches or gum, as part of their treatment plan. This was funded by the local NHS Smoking Cessation Services (Smoking Concerns, Glasgow, UK).

Follow-up information, at 3 and 6 months, was collected via self-completed questionnaires. At one year, data were collected by telephone by a research hygienist.

The outcome of quitting (point prevalence) was measured at 3, 6 and 12 months, with repeated point prevalence measures reported at 6 and 12 months. The measures used included self-report as well as CO and cotinine (COT) measurements. Biochemical verification of smoking status of all participants, whether smokers or non-smokers was undertaken at 3 and 6 months. At 12 months, if a participant reported that they had quit, they were asked to return a sample of saliva by post for verification of smoking status. No cotinine samples were collected from smokers at 12 months. A cut-off of 20 ng/ml cotinine was used to determine a smoker from a non-smoker, whereas a cut-off of 8 ppm was used for the CO monitoring. Information was also collected on use of NRT at the time of data collection.

For those participants who considered themselves smokers at 3 and 6 months, information was collected on the number and length of any quit attempts and current smoking behaviours, in addition to CO and COT samples.

The number of visits by the study participants, to the dental hygienists, during the study period was also collected.

Information from the questionnaires and biochemical information regarding CO and COT levels was entered into data entry forms designed in Microsoft Access, which was then used to manage the data. Data were then exported for statistical analysis into Minitab (version 14) and StatXact (version 4).

Statistical analysis compared the intervention and control groups in terms of baseline information, and outcomes at 3 and 6 months. When comparing the two groups, continuous normally distributed data were summarised by means and standard deviation and analysed using the two-sample t-test and confidence intervals. Data which were numerical but not normally-distributed were summarised by medians and inter-quartile ranges and groups compared using the Mann-Whitney test and confidence intervals. When comparing groups in terms of categorical variables, tables were produced to summarise the data, which were analysed using chi-squared tests. Outcomes, which were binary variables, were compared between groups using tests of equal proportions and confidence intervals for the difference between intervention and control groups. Results of the statistical tests were considered to be significant if the p-value was less than 0.05 and correspondingly if the confidence interval for the average difference between groups did not contain zero.

## Results

**Figure 1:**
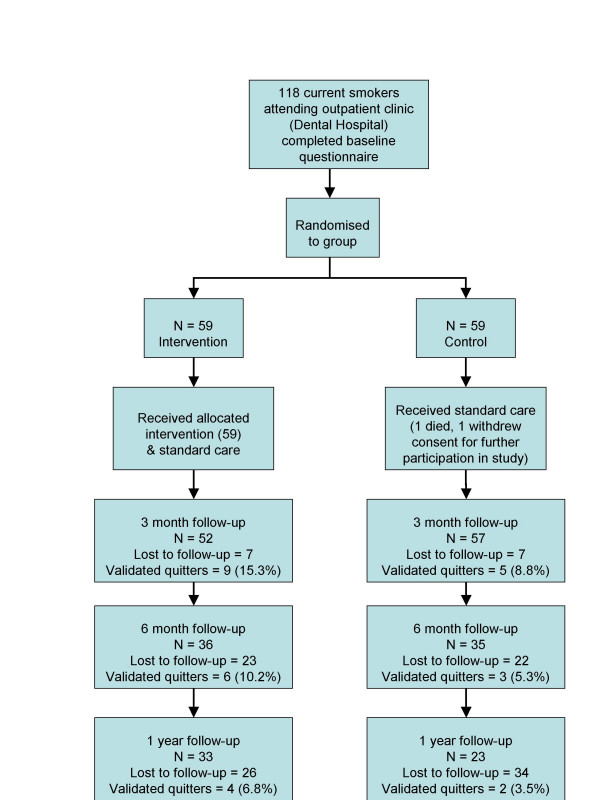
Movement of Participants Through the Study.

At baseline, 118 participants were recruited. One subject died and one subject withdrew consent after completing the baseline questionnaire but prior to attending for any study visits. Therefore data were available for 116 subjects, 59 intervention and 57 control. Figure 1 shows the flow of participants through the trial at the various timepoints.

### Baseline characteristics

There was a predominance of women recruited to the trial (71%). With respect to the baseline characteristics (Table 1), there was no significant difference between intervention and control groups with respect to gender or socio-economic status as measured by DEPCAT. However, there was a statistically significant difference between groups in terms of the mean age of the participants.

**Table 1:** Baseline individual variables at enrolment of periodontal patients

**Variable**	**Intervention (n = 59)**	**Control (n = 57)**	**Comparison of I and C groups**
Demographics			
Mean (SD) Age (years)	39.9 (8.0)	43.5 (8.0)	95% CI (C-I) = (0.6, 6.5)
Gender (female)	45/59 (76%)	37/57 (65%)	χ^2 ^= 1.805 df = 1 p = 0.179
Deprivation Category (5–7 i.e. more deprived)	28/59 (47%)	29/57 (51%)	χ^2 ^= 0.136 df = 1 p = 0.713
**Nicotine Dependence and Exposure**			
Median (IQ) number of cigarettes/day	20 (15–40)	20 (15–25)	95% CI (C-I) = (-1,5)
Mean (SD) number of years smoked	22.5 (7.9)	25.2 (8.2)	95% CI (C-I) = (-0.3, 5.6)
Mean (SD) pack years	21.5 (12.5)	24.8 (12.1)	95% CI (C-I) =(-1.3, 8.0)
Median (IQ) Heaviness of Smoking Index (HSI)	3.0 (3.0–4.0)	4.0 (3.0–4.0)	95% CI (C-I) = (0.0, 1.0)
Median (IQ) Fagerstrom Test for Nicotine Dependence (FTND)	5.0 (3–6)	5.0 (3–7)	95% CI (C-I) = (0.0, 1.0)
Exposure to smoke at home	32/59 (56%)	27/57 (52%)	χ^2 ^= 1.061 df = 2 p = 0.628
Intention to quit			
Motivation to quit/'Stage of Change'			
• Precontemplator	6/59 (10%)	9/57 (16%)	χ^2 ^= 1.076 df = 2 p = 0.584
• Contemplator	26/59 (44%)	26/57 (46%)	
• Preparation	27/59 (46%)	22/57 (38%)	
Biochemical Measures			
Mean (SD) cotinine level (ng/ml)	231.9 (95.0)	243.3 (104.3)	95% CI (C-I) = (-25.3, 48.2)
Mean (SD) CO level (ppm)	22.0 (8.8)	20.8 (9.1)	95% CI (C-I) = (-2.2, 4.5)

There was no statistically significant difference between intervention and control groups with respect to any of the smoking behaviours or nicotine dependence variables, nor was there a significant association between the allocated group and motivation to quit as measured by 'Stage of Change'. With regards to those not interested in quitting, 13% of all trial participants indicated that they were not interested in stopping smoking at the start of the study.

There was no significant difference between the mean baseline biochemical values in the two groups (Table 1).

### Three month outcomes

Data were collected from 102 of 116 participants (87.9%) at this timepoint.

Table 2 outlines the numbers of participants followed up, in addition to further information on quitters as determined by self-report, CO and COT levels, for the two groups.

**Table 2:** Breakdown of number of participants followed-up and quitters at 3 and6 months and 1 year, as defined by self-report, carbon monoxide and salivary cotinine

	**3 months**		**6 months**		**1 Year**	
	**Intervention** ** (n = 59)**	**Control ** **(n = 57)**	**Intervention** ** (n = 59)**	**Control** ** (n = 57)**	**Intervention** ** (n = 59)**	**Control** ** (n = 57)**

Number (%) followed up	52 (88.1%)	50 (87.7%)	36 (61.0%)	35 (61.4%)	33 (55.9%)	23 (40.4%)
Number (%) of self-reported quitters	9/59 (15.3%)	5/57 (8.8%)	6/59 (10.2%)	3/57 (5.3%)	7/59 (11.9%)	3/57 (5.3%)
Number (%) of quitters by Carbon Monoxide	9/59 (15.3%)	4/57 (7.0%) (1 missing value)	6/59 (10.2%)	2/57 (3.5%) (1 missing value)	na	na
Number (%) of quitters by Cotinine	6 + 1* + 2** (15.3%)	2 + 2* + 1** (8.8%)	5 + 1* (10.2%)	2 + 1* (5.3%)	4 + 1^† ^+ 2^‡^	2 + 1^†^
**Quitters**	**9/59 (15.3%)**	**5/57 (8.8%)**	**6/59 (10.2%)**	**3/57 (5.3%)**	**4/59 (6.8%)**	**2/57 (3.5%)**

Carbon monoxide (CO) quitter: <8 ppm, Cotinine (COT) quitter: <20 ng/ml

*COT >20 ng/ml but evidence of NRT use at time of sampling

**COT >20 ng/ml but CO <<8 ppm

^†^self reported quitter but cotinine level above cut-offs (373 ng/ml and 385 ng/ml)

^‡^self reported quitter but did not return biochemical sample

In summary, with respect to all trial participants, the 9 intervention and 5 control participants determined as having quit smoking, represented quit rates of 15.3% and 8.8% respectively. However, the difference between intervention and control groups, in terms of the proportion of participants classified as quitters, did not reach statistical significance (p = 0.449, 95% CI (I-C) = (-8.4, 25.6)%).

In relation to NRT usage, 6/9 quitters in the intervention group had used NRT supplied as part of the study, with 3 using no NRT. In the control group, 1 individual had used Zyban, a further 3 had obtained NRT from another source, such as the general medical practitioner, and one control participant reported that they had not used any form of biochemical support.

For those participants still smoking, a number of outcomes were collected with respect to any changes in smoking and quitting behaviours (Table 3). With regards to quit attempts, at 3 months, there was a statistically significantly higher proportion of participants who reported a sustained quit attempt of one week or longer in the intervention group (16/43; 37%) compared to the control group (8/44; 18%) (p-value = 0.043, 95% CI (I-C) = (1, 37)%). There was also a statistically significantly higher proportion of participants who reported reducing their smoking behaviour in the intervention group (35/43; 81%) compared to the control group (20/44; 45%) (p-value < 0.001, 95% CI (I-C) = (17, 55)%). With regards to other self-reported potential changes, such as inhaling less, or smoking less of a cigarette, there was no statistically significant difference between the groups at this timepoint.

**Table 3:** Self-reported quit attempts (number and duration) and changes insmoking behaviours of participants at 3 months

	**Number (%) of Participants**				
	
**Quit attempts:**	**All **n = 87	**Intervention **n = 43	**Control **n = 44	**p-value**	**95% CI (I-C)**
In last 3 months	43 (49%)	24 (56%)	19 (43%)	0.235	(-8, 34)%
2 or more	27 (31%)	14 (33%)	13 (30%)	0.761	(-16, 23)%
24 hours or more*	40 (47%)	23 (55%)	17 (39%)	0.129	(-5, 37)%
**1 week or more**	**24 (28%)**	**16 (37%)**	**8 (18%)**	**0.043**	**(1, 37)%**
**Other changes:**					
Delay Smoking for >5 min	78 (90%)	40 (93%)	38 (86%)	0.303	(-6, 19)%
Inhale Less of a Cigarette	21 (24%)	12 (28%)	9 (18%)	0.415	(-11, 25)%
Smoke Less of a Cigarette	44 (51%)	24 (56%)	20 (45%)	0.331	(-11, 31)%
Changed to Low Tar Cigarettes	19 (22%)	12 (28%)	7 (16%)	0.172	(-5, 29)%
**Reduced Number of Cigarettes per day**	**55 (63%)**	**35 (81%)**	**20 (45%)**	**<0.001**	**(17, 55)%**

The median number of visits to the staff hygienists between baseline and three months was 4.5 visits (range 1–6) for quitters, with the median number of visits for trial participants categorised as smokers at 3 months being 4.0 (range 2–7).

### Six month outcomes

With respect to the 6 month outcomes, information was collected from 71/116 (61.2%) of the participants.

Of the intervention group, 6 self-reported that they had quit, and CO measurements confirmed this in all cases (Table 2). With respect to cotinine verification, there was evidence that one participant was using NRT, with 5 having cotinine levels below 20 ng/ml. In the control group, 3 self-reported having quit and this was confirmed by CO in 2 of the cases (there was one missing CO value). Cotinine levels confirmed cessation for 2 of the participants, with one person reported as taking NRT. This translates to 6/59 quitters (10.2%) in the intervention group, compared to 3/57 (5.3%) in the control group. However, there was no statistically significant difference between the intervention and control groups in terms of the proportion of participants classified as having quit (p-value = 0.530, 95% CI (I-C) =(-8.5, 23.6)%). With regards to repeated point prevalence, there were 5/59 (8.5%) in the intervention group, compared to 3/57 (5.3%) in the control group (p-value = 0.671, 95% CI (I-C) =(-9.9, 21.8)%).

Characteristics of the 6 month quitters indicated that all were female, with 7 out of 9 coming from the more affluent DEPCAT 1–4 categories.

With regards to NRT use, all successful 6-month quitters used some form of biochemical support at some point in their quit attempt. The intervention participants had used patches supplied as part of the study, one control had used Zyban and two other control individuals had used other manufacturers brands of patch.

As at 3 months, for those participants still smoking, a number of outcomes were collected with respect to any changes in smoking and quitting behaviours (Table 4). At 6 months, with regards to quit attempts, a greater proportion in the intervention group reported a quit attempt of 1 week or more (14/30; 47%), compared to the control group (5/32; 16%). This result was of statistical significance (p-value = 0.018, 95% CI (I-C) = (6, 56)%). There was also a statistically greater proportion of participants in the intervention group who reported any quit attempt in the last three months (25/30; 83%) compared to the control group (18/32; 56%) (p-value = 0.033, 95% CI (I-C) = (3, 53)%). There were no statistically significant differences between the groups, at 6 months, in terms of any other changes in smoking behaviour.

**Table 4:** Self-reported quit attempts (number and duration) and changes insmoking behaviours of participants at 6 months

	**Number (%) of Participants**				
	
**Quit attempts:**	**All **n = 62	**Intervention **n = 30	**Control **n = 32	**p-value**	**95% CI (I-C)**
**In last 3 months**	**43 (69%)**	**25 (83%)**	**18 (56%)**	**0.033**	**(3, 53)%**
2 or more	22 (35%)	13 (43%)	9 (28%)	0.260	(-11, 41)%
24 hours or more	33 (53%)	18 (60%)	15 (47%)	0.330	(-13, 40)%
**1 week or more**	**19 (31%)**	**14 (47%)**	**5 (16%)**	**0.018**	**(6, 56)%**
**Other Changes:**					
Delay Smoking for >5 min	54 (87%)	28 (93%)	26 (81%)	0.380	(-10, 38)%
Inhale Less of a Cigarette	12 (19%)	7 (23%)	5 (16%)	0.546	(-17, 35)%
Smoke Less of a Cigarette	27 (44%)	14 (47%)	13 (41%)	0.688	(-20, 34)%
Changed to Low Tar Cigarettes	21 (34%)	12 (40%)	9 (28%)	0.386	(-14, 38)%
Reduced Number of Cigarettes per Day	38 (61%)	20 (67%)	18 (56%)	0.430	(-14, 38)%

The median number of visits for quitters at 6 months was 7 (range 6–8), while the median number of visits for trial participants categorised as smokers at 6 months was 6 (range 1–8).

### One year outcomes

At one year information was gathered from 48.3% of the participants, 55.9% (33/59) in the intervention group, and 40.4% (23/57) in the control group (Table 2). There were 7 (11.9%) self-reported quitters in the intervention group, with 3 (5.3%) in the control group. Saliva samples for cotinine analysis were received from eight out of ten self-reported quitters. Both individuals who did not return their samples were from the intervention group. In addition, two samples (one from each group) had levels far above the cut-off (373 and 385 ng/ml) and there were no participants who reported using NRT. Therefore the biochemically validated quit rate at one year was 4/59 (6.8%) for the intervention group and 2/57 (3.5%) for the control group. With respect to repeated point prevalence measures at one year, there were 3/59 (5.1%) of participants in the intervention group and 2/57 (3.5%) in the control group who were also quitters at both 3 and 6 months.

## Discussion

This study aimed to determine the feasibility of undertaking a smoking cessation trial utilising trained dental hygienists within a secondary care setting. There is currently little valid research in this area, utilising an RCT design and with the quit rates biochemically validated. The aim was to develop a study protocol utilising an RCT design, with a detailed system of randomisation and testing the 5As model of smoking cessation in a dental setting. In addition, NRT advice and products were delivered by the dental hygienists at a time when the use of such products in hospital periodontal dental practice was not usual. Stringent use of biochemical measures, both cotinine and CO, were a feature of this trial. While cotinine is considered the most accurate method of measuring tobacco exposure, CO is the most widespread means of determining smoking status [20].

With respect to elements of the study protocol, it was possible to recruit patients into a clinical trial with an RCT design. The randomisation, though time consuming to carry out, did produce similar groups with the exception of age. In this trial, the system of randomisation used was by practitioner, with each study hygienist delivering both intervention and control care. This may have led to some contamination, resulting in a higher than expected quit rate in the control group. In a definitive multi-centre study, a cleaner study design would be to randomise by practice, rather than practitioner. A structured protocol utilising the 5As and emphasising the oral health aspects as well as general health benefits, was developed and completed at each visit the patient received smoking cessation advice. The dental hygienists were trained in the use of NRT, and discussed this with the patients who wished to use it during their quit attempt.

There is debate over the most appropriate timeframes to use for follow-up in smoking cessation trials [21]. Researchers are in agreement that the longer the follow-up, the more valid the findings. However, it is also known that the longer the follow-up, the higher the number of participants lost to follow-up. The Cochrane Review of tobacco cessation in a dental setting requires a minimum follow-up of 6 months, and the *Society for Nicotine and Tobacco Research *recommends 6 and 12 month follow-ups [21]. Many studies report a shorter time period of 1 or 3 months, which gives an indication of earlier outcomes including a higher follow-up and success rate. In this trial, while it was possible to collect data from 88% of participants at 3 months, this had fallen to 61% by 6 months and 48% at one year. However, as is standard practice in tobacco cessation work, all patients lost to follow-up can be included in the analysis, by assuming that they are still smoking. Participants by the later time periods had often completed their periodontal treatment and were more difficult to trace, and would often decline to return for follow-up visits, when no treatment was required.

While the number of participants recruited to the trial was relatively small, this was of a similar size to other smoking cessation trials in a dental setting [22-24]. With regards to the cessation rates, the shorter the length of follow-up, the higher the number of participants who stop smoking, and the results of this study reflect this, with a 15.3% quit rate in the intervention group at 3 months, compared to 10.2% at 6 months. The quit rate at one-year in the intervention group was 7.0%. Point prevalence quit rates in the intervention group were approximately twice those in the control group, at each of the three follow-up time points (3, 6 and 12 months). Repeated point prevalence measures showed smaller differences between intervention and control groups at 6 and 12 months. The differences in point prevalence quit rates, however, between the intervention and control groups in this pilot study were not statistically significant. This was, in part, a result of the relatively small number of participants recruited and clearly a larger, statistically powered, RCT study is required to confirm the potential effect of the intervention shown here. Based on the 6-month results (point prevalence) from this preliminary study, a simple sample size calculation showed that to declare statistical significance of a 5% difference (with 10% cessation rate in the intervention group and 5% cessation rate in the control group), with 80% power, would require approximately 465 participants per group to be followed up at 6 months. Audit figures for the department used in this pilot study suggest that there is a throughput of approximately 275 suitable patients per year, and thus it would appear that any future/subsequent RCT would need to be of multi-centre design (with appropriate sample size calculations).

The median number of visits at 6 months was 6–7, higher than one would expect in other dental settings, such as in primary care. Therefore, a success rate of 10% (point prevalence) is modest, verging on disappointing, for the relative amount of time invested. However, half of the participants recruited were from the more deprived areas, as measured by DEPCAT, and it is widely known that the success rate is lower with individuals from more deprived backgrounds [25].

Amongst the group of trial participants who continued to smoke, regarding the outcome of increase in number of quit attempts, at both 3 and 6 month follow-up timepoints there was a statistically higher percentage of individuals in the intervention group compared to the control group who reported that they had made a quit attempt of at least one week. This was a positive behaviour change on the part of the participants. It has been reported that having successful quit attempts, albeit if the patient relapses, helps increase patient efficacy, increasing the likelihood that the patient will be successful in the future [26,27]. With regards to reduction in daily number of cigarettes at 3 months, 81% of the intervention group reported reducing, compared to 45% of the control group (p < 0.001). Currently there is increasing interest in harm reduction strategies such as reduction in daily number of cigarettes, as a precursor to quitting.

Given the lack of good quality research in this area, there is a paucity of studies against which to compare the results of this study. The highest level of research evidence is that of systematic reviews. One review of different types of smoking cessation interventions such as 'brief' opportunistic advice from a physician, or the use of different forms of nicotine replacement therapy, utilises evidence from the Cochrane Library [28]. The intervention most similar in type to the trial reported in this paper, is that of an intervention using NRT and intensive support (defined as an initial session of more than 30 min). The success rate of this type of intervention is quoted as 6% of participants being quitters at 6 months [28]. In this case, success is measured by reporting the difference between the intervention and 'usual care'/control groups. This current trial, using trained dental hygienists to deliver the advice, showed a 5% difference between the quit rates of the intervention and control groups at 6 months which is similar to that of other health professionals [28].

Within the UK, there has been limited tobacco cessation work undertaken in a dental setting. This intervention was delivered in a specialised secondary care setting and its findings may not be generalisable to primary care. A contemporaneous study, undertaken in Newcastle, utilised dental hygienists to give advice in a hospital periodontal department. The Newcastle study yielded a success rate of 29% at 6 months, falling to 25% at one year. This trial had as its main outcome observing the effect of quitting on the periodontium [29]. There has been one trial utilising both dentists and hygienists in primary care, and this resulted in a quit rate of 11% at 9 months [22]. However neither of these trials was of an RCT design. Randomised controlled trials are widely considered to be the most reliable form of scientific evidence because it is the best-known design for the elimination of bias that regularly compromises the validity of research [30].

The dearth of high quality evidence may also be an indicator of how time-consuming tobacco cessation work is to undertake, with large effort expended for relatively little 'gain'. As much effort can be expended undertaking patient follow-ups, as is spent on delivering the cessation advice in the first place, and with relatively small numbers of individuals quitting. However, while gains in the form of quitters may be small with regards to absolute numbers, from a public health perspective, benefit may be large both on a population and individual health gain basis [31].

Another model of care for those patients highly addicted to nicotine, though motivated to stop, is known as the 2As and 1 R [32]. With this model, the advice for dental (as well as medical) practices, is to ask your patient about smoking, advise your patient about the benefits of stopping, and refer those patients most likely to benefit from additional help and support to the specialist stop smoking services. Evaluation of such English services would indicate that 53% of clients were quitters at four weeks[33], falling to 14.6% at one year [34].

However, not all specialist services have developed adequate referral pathways from primary/secondary dental care for patients to be streamed towards specialist services, and there may be patients who have expectations that smoking cessation advice be delivered to them in the dental setting, as part of the whole care package [35]. Dental hygienists, by the nature of their training and role within the dental team, may be the team members best placed to deliver smoking cessation advice. Barriers such as lack of funding and training are often cited [36-38]. The training issues can be dealt with, and a number of initiatives in the UK are attempting to address this [39,40]. Within the US, innovative use of new technologies and more interactive training methods are currently being developed and disseminated [41].

## Conclusion

Staff in the dental setting may well have a role in encouraging their patients towards quitting and helping them, either via referral pathways, or, providing the dental staff are trained and adequately resourced, in delivering the advice themselves [42]. This study has shown the potential that trained dental hygienists could have in delivering smoking cessation advice. However, to add to the definitive knowledge-base, a multi-centred randomised controlled trial, utilising biochemical verification, with the smoking cessation advice delivered by trained dental hygienists, would be required to be undertaken.

## Competing interests

VB has received funding from Pfizer for attending meetings, and has written articles on smoking cessation for GSK. None of the other authors have conflicting interests.

## Authors' contributions

VIB was involved in conception, design and implementation of study, interpretation of the data, and writing and revising the paper. SM was involved in the conception, design and implementation of study, analysis and interpretation of data and drafting of the paper. WB was responsible for the cotinine analysis. WMJ was responsible for advising on the clinical element of the trial, and for enabling the dental hygienists to be involved. LMM was responsible for advice on trial design, for obtaining funding from the dental school for cotinine analysis, and editing the paper. VIB is guarantor. Finally, all authors read and approved the final manuscript.

## Pre-publication history

The pre-publication history for this paper can be accessed here:


